# A Nordic survey of CT doses in hybrid PET/CT and SPECT/CT examinations

**DOI:** 10.1186/s40658-019-0266-7

**Published:** 2019-12-16

**Authors:** Natalie A. Bebbington, Bryan T. Haddock, Henrik Bertilsson, Eero Hippeläinen, Ellen M. Husby, Virpi I. Tunninen, Marcus Söderberg

**Affiliations:** 1 Siemens Healthcare A/S, Bredskifte Alle 15, 8210 Aarhus, Denmark; 2 Department of Clinical Physiology, Nuclear Medicine and PET, Rigshospitalet, Copenhagen University Hospital, Ndr. Ringvej 57, 2600 Glostrup, Denmark; 3 Siemens Healthcare AB, Evenemangsgatan 21, 16979 Solna, Sweden; 4 HUS Medical Imaging Center, Clinical Physiology and Nuclear Medicine, Nuclear Medicine Unit, Meilahti Hospital, University of Helsinki and Helsinki University Hospital, Tukholmankatu 8 F, PL 442, 00029 HUS Helsinki, Finland; 5 Department of Diagnostic Physics, Gaustad Hospital, Oslo University Hospital, Building 20, P.O. Box 4959, N-0424 Nydalen, Oslo Norway; 6 Department of Nuclear Medicine, Satakunta Central Hospital, Sairaalantie 3, 28500 Pori, Finland; 7 Medical Radiation Physics, Department of Translational Medicine, Lund university, Inga Marie Nilssons gata 49, 20502 Malmö, Sweden; 8 Radiation Physics, Department of Hematology, Oncology and Radiation Physics, Skåne University Hospital, Inga Marie Nilssons gata 49, 20502 Malmö, Sweden

**Keywords:** Computed tomography, Diagnostic reference levels, Dosimetry, Hybrid imaging, PET/CT, SPECT/CT

## Abstract

**Background:**

Computed tomography (CT) scans are routinely performed in positron emission tomography (PET) and single photon emission computed tomography (SPECT) examinations globally, yet few surveys have been conducted to gather national diagnostic reference level (NDRL) data for CT radiation doses in positron emission tomography/computed tomography (PET/CT) and single photon emission computed tomography/computed tomography (SPECT/CT). In this first Nordic-wide study of CT doses in hybrid imaging, Nordic NDRL CT doses are suggested for PET/CT and SPECT/CT examinations specific to the clinical purpose of CT, and the scope for optimisation is evaluated. Data on hybrid imaging CT exposures and clinical purpose of CT were gathered for 5 PET/CT and 8 SPECT/CT examinations via designed booklet. For each included dataset for a given facility and scanner type, the computed tomography dose index by volume (CTDI_vol_) and dose length product (DLP) was interpolated for a 75-kg person (referred to as CTDI_vol,75kg_ and DLP_75kg_). Suggested NDRL (75th percentile) and achievable doses (50th percentile) were determined for CTDI_vol,75kg_ and DLP_75kg_ according to clinical purpose of CT. Differences in maximum and minimum doses (derived for a 75-kg patient) between facilities were also calculated for each examination and clinical purpose.

**Results:**

Data were processed from 83 scanners from 43 facilities. Data were sufficient to suggest Nordic NDRL CT doses for the following: PET/CT oncology (localisation/characterisation, 15 systems); infection/inflammation (localisation/characterisation, 13 systems); brain (attenuation correction (AC) only, 11 systems); cardiac PET/CT and SPECT/CT (AC only, 30 systems); SPECT/CT lung (localisation/characterisation, 12 systems); bone (localisation/characterisation, 30 systems); and parathyroid (localisation/characterisation, 13 systems). Great variations in dose were seen for all aforementioned examinations. Greatest differences in DLP_75kg_ for each examination, specific to clinical purpose, were as follows: SPECT/CT lung AC only (27.4); PET/CT and SPECT/CT cardiac AC only (19.6); infection/inflammation AC only (18.1); PET/CT brain localisation/characterisation (16.8); SPECT/CT bone localisation/characterisation (10.0); PET/CT oncology AC only (9.0); and SPECT/CT parathyroid localisation/characterisation (7.8).

**Conclusions:**

Suggested Nordic NDRL CT doses are presented according to clinical purpose of CT for PET/CT oncology, infection/inflammation, brain, PET/CT and SPECT/CT cardiac, and SPECT/CT lung, bone, and parathyroid. The large variation in doses suggests great scope for optimisation in all 8 examinations.

## Background

Computed tomography (CT) was first made commercially available on a hybrid single photon emission computed tomography/computed tomography (SPECT/CT) system in 1999 and on a positron emission tomography/computed tomography (PET/CT) system in 2000 [1] and use of hybrid molecular imaging (MI) CT systems has since grown rapidly [1, 2]. In 2016, there were almost a thousand PET/CT scanners available in European Union (EU) member states, derived from figures provided by Eurostat [3] and United Kingdom (UK) figures from Dickson and Eve [4], and there were at least 193 SPECT/CT scanners in the UK alone [2]. The rapid diffusion and utilisation of PET/CT and SPECT/CT scanners may raise concern for patient radiation exposure [5–7]. Therefore, nuclear medicine professions should optimise CT radiation doses following the as low as reasonably achievable (ALARA) concept.

Diagnostic reference levels (DRLs) allow facilities to evaluate their practice, by comparing radiation doses given locally with those given in the wider population. National DRLs (NDRLs) are published by relevant radiation authorities, which are informed in the first instance by collection of radiation dose data. The use of DRLs enable the health professions to compare their third quartile values for the radiation dose measures of computed tomography dose index by volume (CTDI_vol_) and dose length product (DLP) against the NDRL standard for their respective country. Third quartile values are often supplemented with median values as achievable doses, which serve as an additional reference level to aid optimisation [8].

Establishing CT DRLs specifically for hybrid imaging is complicated by the clinical purpose of the CT scan, which could be for *attenuation correction (AC)* of the SPECT or PET signal; anatomical *localisation* of increased or reduced tracer uptake in the MI images; *characterisation* from the CT scan of the disease aetiology of abnormal tracer uptake seen on the MI images; or fully *diagnostic* purposes, where the exposure settings are equivalent to those used in standalone CT protocols in the radiology department. The image quality and therefore radiation doses required for the different clinical purposes increase from AC only through to fully diagnostic. Thus, when using information on radiation doses or exposure settings for reference, this should relate to the same clinical purpose [2].

Use of DRLs for common examinations involving ionising radiation has been required in the European Medical Exposures Directive for more than 20 years [9]. However, there are to date no published NDRLs for CT in hybrid imaging for the vast majority of countries worldwide. CT radiation doses for PET/CT oncology whole body examinations are the most widely investigated of all MI examinations, with national surveys having been conducted in France [10], Bulgaria [11], United States of America (USA) [12, 13], Korea [14], UK [2], Switzerland [15], and Australia and New Zealand [16]. Meanwhile, CT dose data for SPECT/CT and other PET/CT examinations is more sparse, with national surveys for SPECT/CT having only been conducted in the UK [2], Switzerland [15], and Bulgaria [17]. These studies have shown large diversity amongst facilities in the clinical purpose of CT for the same examination [2], and in mean radiation doses given by different facilities for the same examination and clinical purpose, which demonstrates a great need for optimisation for CT in hybrid imaging [2, 10, 14]. These studies have also demonstrated large differences in doses given by different countries for the same examination and clinical purpose [2], which highlights the need for country- or region-specific reference data. The International Commission on Radiological Protection (ICRP) states that DRL values should be used for reporting radiation doses for PET, SPECT, and CT components of nuclear medicine hybrid imaging examinations [18]. However, since Denmark, Finland, Norway, and Sweden have already established country-specific NDRLs for administered radiopharmaceutical activities [19–22], this survey does not focus on administered radiopharmaceutical activities. This Nordic-wide multi-centre study rather focuses on gathering reference data for the CT component of PET/CT and SPECT/CT examinations.

This survey aims to suggest a Nordic NDRL for CT doses associated with PET/CT and SPECT/CT scans. It further evaluates the scope for optimisation by assessing variation in doses between facilities in the Nordics.

## Methods

### Overview

Dose data has been gathered for CT scans undertaken for 5 PET/CT examinations (oncology, infection/inflammation, brain, cardiac (myocardial perfusion), and bone) and 8 SPECT/CT examinations (cardiac (myocardial perfusion), lung, bone, parathyroid, sentinel node, octreotide, metaiodobenzylguanidine (mIBG), and thyroid post ablation). This study also controls for confounding variables present in some previous studies, by controlling for differences in body mass within the population as recommended by the ICRP [18].

### Ethics approval and consent to participate

This study is exempt from notification to a research ethics committee under Section 14 of the Danish *Act on Research Ethics Review of Health Research Projects* [23].

### Data collection

Each facility in Denmark, Finland, Norway, and Sweden undertaking PET/CT and/or SPECT/CT examinations was invited to participate in the study. For each type of examination, a data capture form requested information on the following: scanner type; clinical purpose of CT (AC only, localisation, characterisation, or fully diagnostic); protocol settings; and data for up to 30 patients (Additional file 1).

### CT protocol

Requested data included acquisition and reconstruction settings in the protocol influencing image quality and dose, and use of dose optimisation features such as tube current (mA) modulation, tube voltage (kV) selection, and iterative reconstruction. As the purpose of this study was to establish suggested NDRL CT doses for MI-specific practices, facilities were asked to submit data for fully diagnostic purposes only in cases where the fully diagnostic scan was the only CT scan undertaken.

### Patient data

Requested data included sex of patient, height, body mass, body region scanned, and radiation dose reported by the scanner (CTDI_vol_, DLP). Data were collected between April 2017 and February 2018.

### Exclusion criteria

For protocols utilising tube current modulation, patient datasets were excluded if body mass was not recorded or if data were recorded for less than 10 patients (as this would not be sufficient to provide reliable data for weight-derivation for a 75-kg person).

### Data analysis

All data analyses were undertaken using Microsoft Excel (Microsoft Office 365 Pro Plus). For each system-examination combination where tube current modulation was used, measured CTDI_vol_ and DLP values for patients were plotted against their body mass. The GROWTH function was then used to calculate the predicted exponential growth and fit an exponential curve to the existing *x*- and *y*-values. From this curve, a single dose value (CTDI_vol,75kg_ and DLP_75kg_) for a 75-kg patient was interpolated/extrapolated for that system-examination combination. This was used for all examinations, except PET/CT brain where the mean value was used. For data submissions from systems where the scans did not apply tube current modulation, mean CTDI_vol_ and DLP values were used. Mean scan length was estimated for each system for each examination by dividing mean DLP by mean CTDI_vol_. Quantitative CTDI_vol,75kg_, DLP_75kg_, and scan length values for each examination and clinical purpose were expressed as mean, median, minimum, maximum, and minimum/maximum. NDRL (75th percentile) and achievable doses (50th percentile) were suggested for PET/CT and SPECT/CT examinations specific to the clinical purpose of CT, where there were data submissions for 10 or more systems.

Cardiac SPECT and PET data were combined, as were octreotide and mIBG. Data for scans covering only the head or extremities were separated from data for scans covering the *main body* (shoulders, thorax, abdomen, pelvis) where there is more attenuation. For PET/CT oncology, in cases where multiple CT scans were performed for a single patient, data were analysed for the standard vertex to mid-thigh scan range, with additional diagnostic scans removed from the analysis. However, presented diagnostic data for the vertex to mid-thigh range may contain more than one scan phase where intravenous contrast is used.

Brain PET/CT data were normalised to use of a 16-cm diameter CTDI phantom for each CT system included in the study, allowing a valid comparison between different systems. The conversion factors between 16- and 32-cm diameter CTDI phantoms were calculated as CTDI_vol_ for the 16-cm phantom divided by CTDI_vol_ for the 32-cm phantom, based on data from each scanner’s system specifications.

To understand the main sources of difference in dose between facilities for the same examination and clinical purpose of CT, where possible, protocol settings were compared between the systems giving the maximum and minimum DLP_75kg_ for a given examination and clinical purpose. In order to make fair comparisons, it was necessary to use *effective mAs*, which is a measure of photon flux that accounts for the influence of tube rotation time and pitch factor. Effective mAs is calculated as follows:

$$ \left(\mathrm{tube}\ \mathrm{current}\ \left(\mathrm{mA}\right)\ast \mathrm{tube}\ \mathrm{rotation}\ \mathrm{time}\ \left(\mathrm{s}\right)\right)/\mathrm{pitch}\ \mathrm{factor} $$

## Results

Data were processed from 83 different scanners submitted from a total of 43 Nuclear Medicine facilities. This comprised data from 34 PET/CT scanners from 29 facilities (Denmark 13, Finland 5, Norway 6, Sweden 5) and 49 SPECT/CT scanners from 40 facilities (Denmark 13, Finland 6, Norway 11, Sweden 10). This represented a response rate of 76% of Danish Nuclear Medicine facilities, 35% of Finnish facilities, 53% of Norwegian facilities, and 36% of Swedish facilities.

**Fig. 1:**
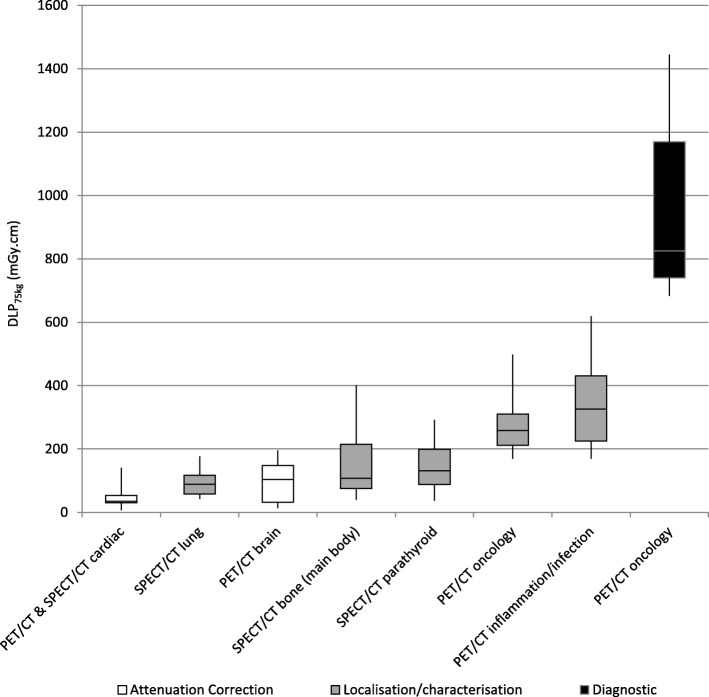
Boxplot showing spread in DLP_75kg_ data for reliable PET/CT and SPECT/CT datasets (Mean DLP data (not weight-derived) normalised to a 16-cm CTDI phantom are given for PET/CT brain)

Figure 1 provides an overview of minimum, 25th percentile, 50th percentile, 75th percentile, and maximum DLP_75kg_ for examination types and clinical purposes with enough data submissions (from 10 or more systems) for reliable interpretation. This demonstrates large variation in dose, both within an examination type and clinical purpose, and between different examinations. For PET/CT oncology, there was diverse practice with some facilities performing extra scan ranges in addition to the standard vertex to mid-thigh CT scan. However, data are presented only for the standard scan length. Data for extra CT scans were removed and contribute an additional radiation dose.

Table 1 presents suggested NDRLs (75th percentile) and achievable doses (50th percentile) for CTDI_vol, 75kg_ and DLP_75kg_ for the examination types and clinical purposes with data submissions from 10 or more systems, with the exception of diagnostic CT in PET/CT oncology where standalone diagnostic NDRLs apply.

**Table 1:** Suggested NDRL and achievable doses for PET/CT and SPECT/CT examinations according to clinical purpose (Mean DLP data (not weight-derived) normalised to a 16-cm CTDI phantom are given for PET/CT brain; NDRL and achievable doses are not presented for PET/CT oncology diagnostic CT scans as standalone diagnostic NDRLs should be used for comparison)

Examination	Clinical purpose	Suggested NDRL (75th percentile)		Achievable dose (50th percentile)	
		CTDI_vol_ (mGy)	DLP (mGy.cm)	CTDI_vol_ (mGy)	DLP (mGy.cm)
PET/CT oncology	Localisation/characterisation	2.9	310	2.6	258
PET/CT infection/inflammation	Localisation/characterisation	3.5	431	2.5	326
PET/CT brain	AC only	6.4	148	5.7	104
PET/CT and SPECT/CT cardiac	AC only	2.2	53	1.6	35
SPECT/CT lung	Localisation/characterisation	2.9	117	2.5	89
SPECT/CT bone (main body)	Localisation/characterisation	4.0	215	2.0	108
SPECT/CT parathyroid	Localisation/characterisation	5.7	199	3.5	131

Table 2 provides summary data (mean, median, maximum (max), minimum (min), and max/min) for CTDI_vol, 75kg_, DLP_75kg_, and scan length, and shows up to 27 times difference in dose between facilities for the same examination and clinical purpose of CT.

**Table 2:** Summary data for CTDI_vol_, _75kg_, DLP_75kg_, and scan length, for all examinations and clinical purposes (Mean DLP data (not weight-derived) normalised to a 16cm CTDI phantom are given for PET/CT brain)

Examination	Clinical purpose of CT	Number of datasets	CTDI_vol,75kg_ (mGy)					DLP_75kg_ (mGy.cm)					Scan length (cm)				
			Mean	Median	Min	Max	Max/min	Mean	Median	Min	Max	Max/min	Mean	Median	Min	Max	Max/min
PET/CT oncology	AC only	6	1.4	1.4	0.3	2.4	8.0	141	136	29	263	9.0	102	103	87	114	1.3
	L/C	15	2.7	2.6	1.8	4.9	2.7	270	258	169	498	2.9	99	99	80	116	1.5
	Diagnostic^a^	11	9.9	8.5	6.4	14.8	2.3	963	825	683	1445	2.1	100	98	85	124	1.5
PET/CT infection/inflammation	AC only	4	1.8	1.4	0.3	4.4	14.7	194	131	27	488	18.1	101	98	95	113	1.2
	L/C	13	3.3	2.5	1.8	7.0	3.9	346	326	169	619	3.7	110	104	89	167	1.9
	Diagnostic	1	8.5	-	-	-	-	766	-	-	-	-	90	-	-	-	-
PET/CT brain	AC only	11	4.2	5.7	0.5	8.5	17.0	94	104	13	196	15.0	22	23	17	25	1.5
	L/C	9	15.0	10.7	3.1	66.9	21.6	302	211	71	1194	16.8	22	23	18	25	1.4
	Diagnostic	1	45.8	-	-	-	-	1068	-	-	-	-	23	-	-	-	-
PET/CT and SPECT/CT cardiac	AC only	30	2.1	1.6	0.4	7.1	17.8	40	35	5	98	19.6	23	23	14	33	2.4
	L/C	7	2.7	1.9	1.3	8.0	6.2	67	39	28	157	5.6	20	20	19	21	1.1
SPECT/CT lung	AC only	2	5.7	5.7	0.4	11	27.5	185	185	13	356	27.4	34	34	32	36	1.1
	L/C	12	2.6	2.5	1.1	5.6	5.1	93	89	42	177	4.2	36	35	30	43	1.5
SPECT/CT bone	AC only	1	2.1	-	-	-	-	235	-	-	-	-	111	-	-	-	-
	L/C	30	2.8	2.0	1.1	7.3	6.6	153	108	40	402	10.0	55	43	37	114	3.1
	Diagnostic	1	2.34	-	-	-	-	255	-	-	-	-	69	-	-	-	-
SPECT/CT parathyroid	AC only	1	3.2	-	-	-	-	100	-	-	-	-	32	-	-	-	-
	L/C	13	4.1	3.5	1.5	10.6	7.1	150	131	37	292	7.8	40	40	24	62	2.6
	Diagnostic	1	15.6	-	-	-	-	365	-	-	-	-	24	-	-	-	-

^a^NDRL and achievable doses are not presented for PET/CT oncology diagnostic CT scans as standalone diagnostic NDRLs should be used for comparison

**Fig. 2:**
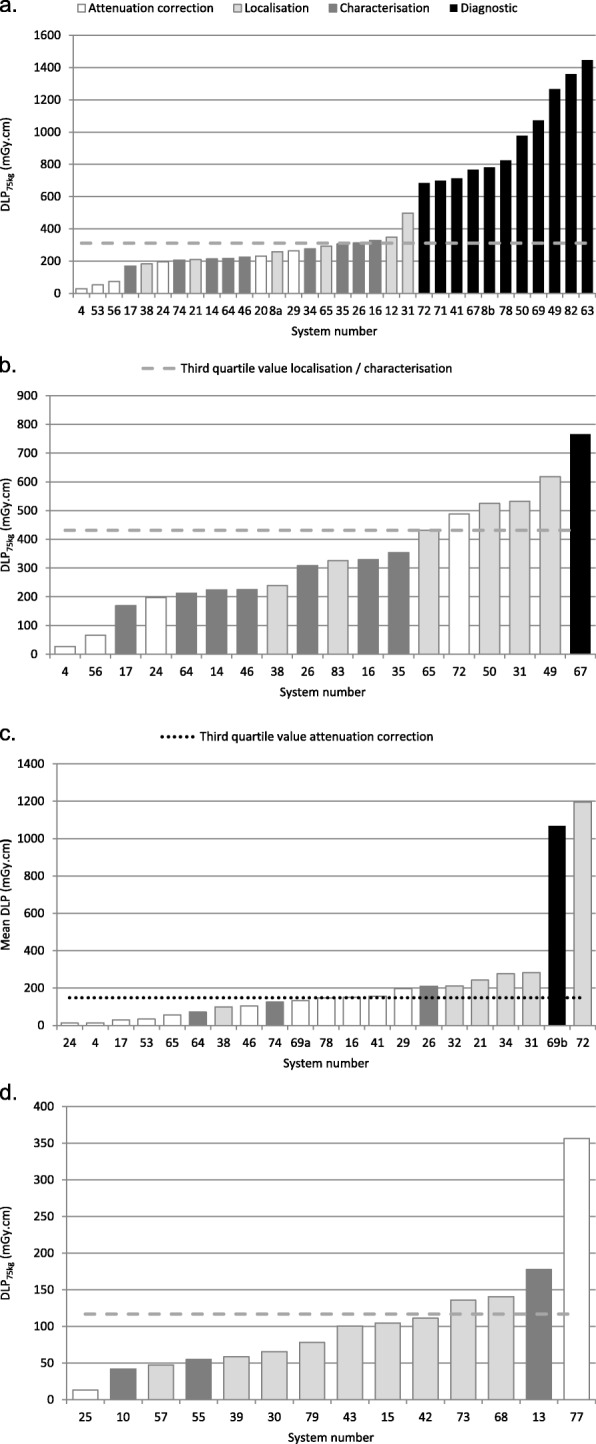
Distribution of DLP_75kg_ according to system number and clinical purpose for PET/CT oncology whole body (Data submissions from a system for more than one clinical purpose are labelled with a letter suffix) (**a**), PET/CT infection/inflammation (**b**), PET/CT brain (Mean DLP data (not weight-derived) normalised to a 16-cm CTDI phantom are given for PET/CT brain; Data submissions from a system for more than one clinical purpose are labelled with a letter suffix) (**c**). SPECT/CT lung (**d**)

Figure 2a to d show the distributions of DLP_75kg_ for included systems according to clinical purpose of CT for PET/CT oncology, PET/CT infection/inflammation, PET/CT brain, and SPECT/CT lung, demonstrating interspersion of the different clinical purposes across the distributions, with AC only doses exceeding suggested NDRLs for localisation/characterisation (Fig. 2b and d) and one system’s localisation dose exceeding another system’s diagnostic dose (Fig. 2c). Given the great dose variations between facilities for the same examination and clinical purpose of CT, Table 3 provides a comparison of protocol settings known to be major contributors to differences in dose, between the systems with the maximum and minimum DLP_75kg_, for the examinations and clinical purposes where comparisons were possible.

**Table 3:** Comparison of key protocol settings contributing to dose differences between maximum and minimum DLP_75kg_ systems

Examination	Clinical purpose of CT	Max/min DLP_75kg_	Max or min DLP_75kg_ protocol	Scan length (cm)	Summary of main protocol differences contributing to variation in DLP_75kg_
PET/CT brain	Localisation/characterisation	16.8	Max	18	Exceptionally high effective mAs for localisation/characterisation (images originally intended for diagnostic use)
			Min	23	
PET/CT and SPECT/CT cardiac	AC only	19.6	Max	29	High effective mAs for AC only; long scan length
			Min	16	Attempted to optimise for AC only with low effective mAs (low mA, high pitch) and low kV; use of IR
SPECT/CT lung	AC only	27.4	Max	32	High kV; high effective mAs for AC only
			Min	36	Attempted to optimise for AC only with low effective mAs (low mA, high pitch); use of IR
	Localisation/characterisation	4.2	Max	32	Higher kV and effective mAs than minimum DLP_75kg_ protocol
			Min	33	Low kV and effective mAs
SPECT/CT bone	Localisation/characterisation	10.0	Max	106	Three times greater scan length, higher kV and effective mAs than minimum DLP_75kg_ protocol
			Min	37	Use of IR
SPECT/CT parathyroid	Localisation/characterisation	7.8	Max	28	Much higher effective mAs (but lower kV) than minimum DLP_75kg_ protocol
			Min	24	

Seventy percent of facilities undertaking PET/CT infection/inflammation examinations used the same CT protocol as that used for their PET/CT oncology protocol whereas 17% used a different clinical purpose of CT, 4% used a different scanner, and in 9% protocol settings were not provided.

## Discussion

This study has suggested Nordic NDRL and achievable doses for CT scans performed in 4 PET/CT and 4 SPECT/CT examinations which are specific to the clinical purpose of the CT scan, as shown in Table 1. The data presented in this study demonstrates great variation in CT radiation doses for the same examination and clinical purpose of CT, for all investigated PET/CT and SPECT/CT examinations, as shown in Fig. 1 and Table 2. For instance, up to 9 times difference in DLP_75kg_ was seen for AC only in PET/CT oncology, with some AC only doses exceeding the achievable dose and approaching the suggested NDRL for localisation/characterisation as shown in Fig. 2a. An 18 times difference in DLP_75kg_ was seen for AC only in PET/CT infection/inflammation, with Fig. 2b showing a system giving AC only doses exceeding the suggested NDRL for localisation/characterisation. For PET/CT brain, Fig. 2c shows a system giving localisation doses greater than a system giving diagnostic doses, constituting a 17 times difference in DLP_75kg_ for localisation/characterisation. In the case of SPECT/CT, up to 27 times difference in DLP_75kg_ was seen for AC only CT scans in lung SPECT/CT, with a system giving AC only doses four times greater than the suggested NDRL for localisation/characterisation, as shown in Fig. 2d. On comparing scan protocol settings for the maximum and minimum dose protocols (for the same examination and clinical purpose of CT) in Table 3, variations in kV, effective mAs, scan length, and reconstruction algorithm were found to be key contributors to dose differences. Such findings thus demonstrate the importance of optimising CT scan protocol settings to provide image quality that is specific to the clinical purpose of the CT scan, and applying available dose optimisation features where appropriate.

PET/CT oncology gives one of the greatest CT radiation burdens of all MI examinations, whether performed for diagnostic or localisation/characterisation purposes, as shown in Fig. 1. It is therefore not surprising that it has been the most widely investigated MI examination in terms of CT radiation dosimetry, with several national CT dose surveys published in the literature, namely from France [10], Bulgaria [11], USA [12, 13], Korea [14], UK [2], Switzerland [15], and Australia and New Zealand [16]. Excessive variation in dose between facilities was also noted for PET/CT oncology examinations in France [10], Korea [14], the UK [2], and Australia and New Zealand [16], suggesting great scope for optimisation globally. This Nordic data presents the lowest localisation/characterisation CT doses for PET/CT oncology examinations published in the literature, with third quartile DLP_75kg_ less than half the value published for France [10]. This highlights the importance of using DRLs which are country- or region-specific. The large dose differences with France could reflect that the data for this study was gathered 6 years later, during which time scanners with more sophisticated dose saving technologies may have been utilised, and there may have become a greater awareness of the need for optimisation. This highlights the need to revise NDRLs every 3–5 years [18]. The slightly lower doses compared with the UK may be a result of the Nordic data being weight-derived for a 75-kg person, whilst the UK study did not apply a weight restriction or a weight-derivation due to lack of submitted data on patient weights.

A possible barrier to facilities performing diagnostic CT scans on hybrid systems could be a concern that scanner technologies could be inferior on hybrid systems to those in the radiology department and doses may be higher. Yet, this study demonstrated that diagnostic CT performed as part of PET/CT oncology examinations is common in the Nordics, and that the third quartile CTDI_vol,75kg_ (12.5 mGy), which comprises data mostly from Danish facilities, is within the third quartile NDRL set by the Danish radiation protection authority (Sundhedsstyrelsen, Statens Institute for Strålebeskyttelse (SIS)) in 2015 for thorax/abdomen examinations (17 mGy) [24]. Diagnostic CT doses for PET/CT oncology presented in this study are not suggested NDRL CT doses, as the countries’ already existing NDRLs for standalone diagnostic CT should be used.

Figure 1 shows that other MI examinations can give CT radiation doses in the same range as PET/CT oncology localisation/characterisation scans. Yet, there are still limited CT dose surveys for other PET/CT and SPECT/CT examinations, with national surveys having only been conducted for other examinations in the UK [2], Switzerland [15], and Bulgaria [17]. Iball et al. noted large variations in CT doses for other PET/CT and SPECT/CT examinations in the UK [2] suggesting a need for optimisation. It is therefore important to also survey CT doses in other PET/CT and SPECT/CT examinations, and all ionising radiation exposures should be optimised in keeping with the ALARA principle.

For PET/CT and SPECT/CT cardiac, median and third quartile CTDI_vol,75kg_ values in this Nordic study are comparable with those in UK [2] and Swiss [15] studies, as is median DLP_75kg_. Yet third quartile DLP values in the Nordic study are considerably greater, representing a greater spread in CT scan length where mean and maximum scan lengths were 23 cm and 33 cm as shown in Table 2, compared with 18 cm and 24 cm in the UK study [2]. Presented dose values represent one CT scan, although patients may have two CT scans as part of the complete test (stress and rest).

Nordic main body doses for SPECT/CT bone scans are in broad agreement with UK data [2], although a tendency for a longer CT scan length is noted in the Nordics. Bone SPECT/CT examinations can cover any body region depending on the patient’s clinical indications. Data were therefore categorised into main body, head, and extremities. It would have been ideal to categorise main body data further according to the specific body part as done in the Swiss survey [15], but the amount of submitted data was insufficient for this. For SPECT/CT parathyroid, Nordic doses are in general agreement with those from the UK [2] and Switzerland [15]. However, a greater CT scan length is noted for Nordic protocols, giving slightly higher DLP_75kg_ than other published values. This is the first study to suggest NDRL CT doses for PET/CT brain and SPECT/CT lung and thus, there are no reliable published dose values for comparison. It is also the first study to suggest NDRL CT doses for PET/CT infection/inflammation. However, the results from this survey showed that the vast majority of facilities used the same CT protocol as for PET/CT oncology, thus suggesting that facilities outside of the Nordics wishing to evaluate their local DRL CT doses for PET/CT infection/inflammation could use their NDRLs for PET/CT oncology as a reference, in the absence of NDRL CT doses specifically for PET/CT infection/inflammation.

In this study, reported AC only radiation doses had the greatest variation in dose. Good image detail is not required for AC only; therefore, very low dose settings can be used to provide enough information for a reliable CT-based attenuation map. Hence, very low dose scans are used in some facilities for AC only, whereas standard diagnostic scanner protocols which have not been optimised for AC only have been used in other facilities. Thus, dose variations tend to reduce from AC only purposes through to diagnostic purposes, with the highest maximum/minimum dose difference of 27 times being for AC only in lung SPECT/CT, and the lowest maximum/minimum dose difference of 2.1 times being for diagnostic PET/CT oncology.

Collecting information on CT protocol settings allows further investigation of dose differences between systems. Table 3 shows that the factors contributing to the 27 times difference in dose for AC only lung SPECT/CT include a higher tube voltage and effective mAs for the protocol providing the maximum DLP_75kg_, compared with a low effective mAs (afforded by low mA and high pitch factor) and use of iterative reconstruction in the lowest dose protocol. A 21 times difference in dose for AC only cardiac PET/CT and SPECT/CT protocols was generated by a very high effective mAs and very long scan length for the heart, in the protocol providing the maximum DLP_75kg_, compared with a low kV, low effective mAs (comprising low mA and high pitch factor), and use of iterative reconstruction in the lowest dose protocol. For PET/CT brain, the main contributor to the 17 times difference in localisation/characterisation dose was an extremely high mAs from the maximum dose protocol compared with other localisation/characterisation protocols, as it was reported that images were originally intended for fully diagnostic purposes, but following a change in circumstance, the images were only being used for localisation in practice. Further protocol comparisons are made in Table 3.

Mattsson et al. [25] described how dose-saving features such as tube current modulation, choice of x-ray spectra, iterative reconstruction, and new detectors have the potential to reduce dose considerably. As the type and availability of these features differ between systems, there will inevitably be dose differences. However, the technical capabilities of the systems alone cannot account for all differences in dose seen in this study. Table 3 demonstrates that no single parameter is causing the large differences in dose for all examinations, and the large factors of difference are being generated through some facilities having multiple parameters which they have tried to optimise for clinical purpose, and other facilities having multiple parameters which are not optimised for clinical purpose, and for some examinations may have selected standard diagnostic protocols for AC only and localisation/characterisation scans. Yet, even where efforts have been made to optimise CT protocols for clinical purpose, there will inevitably be differences in reader preferences for noise and resolution, causing variability in parameters and thus dose.

Differences in scan length for the same examination and clinical purpose of CT also contribute to differences in DLP. CT scan lengths for PET examinations are generally standardised, due to the technical phenomenon that PET/CT systems require the attenuation information gleaned from the CT data for the full PET FOV, in order to allow AC of the PET images, because recorded PET photons are not collimated when scanning in the conventional 3D mode [26]. However, given that recorded SPECT photons are collimated, the CT scan can be localised to the anatomical area of interest, whilst still allowing AC SPECT reconstructions over that area [27]. Although scan length does contribute to dose differences in all scans, it was not considered a major contributor to DLP_75kg_ differences for PET/CT oncology, PET/CT brain, and SPECT/CT lung, contributing to less than 1.5 times difference in dose. However, Table 2 shows that scan length differed markedly for some SPECT/CT examinations, with up to 2.4, 2.6, and 3.1 times difference in scan length for PET/CT and SPECT/CT cardiac (AC only), SPECT/CT parathyroid (localisation/characterisation), and SPECT/CT bone (localisation/characterisation) scans respectively. The difference in cardiac SPECT/CT scan length can be explained by some facilities restricting the CT scan to the heart, whereas others scan a much greater area. Parathyroid adenomas are most commonly located around the thyroid bed but can occasionally be ectopic (sublingual region down to the heart) [28]. Therefore, some facilities localise the SPECT/CT scan to the thyroid region whilst another scans 2 fields-of-view (FOV) to cover the full possible ectopic area. For SPECT/CT bone, some facilities perform 3 FOV SPECT/CT scans (head to thigh) as standard without planar whole body imaging, as SPECT/CT is known to have greater sensitivity and specificity than planar imaging [29], whereas other facilities perform planar whole body gamma camera imaging as standard and supplement this with SPECT/CT over areas of particular interest. The wide variations in scan length for these three examinations are also consistent with the tendency for the Nordic scan lengths shown in Table 2 to be greater than corresponding UK scan lengths [2]. These findings suggest that scan length could be a focus area for optimisation efforts in Nordic SPECT/CT examinations.

Design of a suitable method for reporting CT NDRL doses for CT in MI examinations is essential to enable accurate data comparisons. Many methodological questions arose during the preprocessing of collected data which were difficult to predefine before starting the study. One such source of variability is the clinical purpose of the CT scan. The UK survey grouped data into 3 clinical purposes of CT (attenuation correction, localisation, and fully diagnostic) [2]. This study included a fourth category of characterisation, which should in theory give more detail and thus a higher dose than localisation, but less than diagnostic. However, some facilities communicated that they were not familiar with this term and the data showed no clear distinction in dose between localisation and characterisation. Thus, localisation and characterisation purposes were combined, thereby allowing a greater data pool for generating suggested NDRL CT doses. Furthermore, the validity of these survey results is reliant on the correct clinical purpose of CT being recorded on the data capture form. It is expected that this has been discussed between the relevant health professionals for each facility.

Alkhybari et al. published recommendations for establishing PET/CT and SPECT/CT NDRLs in 2018 [7] after this study had commenced, explaining that future PET/CT and SPECT/CT NDRL data should include a minimum of 50 patients without weight restriction, based on the current ICRP publication [18]. However, this study, similar to that by Iball et al. [2], is limited by the quantity of data submitted. In both studies, data submissions from a facility for a scanner-examination combination were included if there were data for ten or more patients. This is less than the number recommended by the ICRP [18]. However, given that hybrid examinations have a much longer examination time than standalone CT and submitted data must be further subdivided according clinical purpose, it is difficult to obtain as high a number of data submissions compared with standalone CT. Given these limitations on patient numbers, data were acquired in this study without weight restriction to obtain as much data as possible, but since the data included a maximum of 30 patients per system, doses were then interpolated to a 75-kg person to get a more fair comparison. Alkhybari et al. explained that less than 2% difference in dose has been found between weight-restricted or non-weight-restricted methods, meaning that non-weight-restricted methods are still valid [7]. Data were commonly excluded in this study due to insufficient numbers of patients (less than 10 for systems utilising tube current modulation) due to limited throughput during the data collection period. Other reasons for exclusion included absence of patient body mass data meaning that the data could not be weight-derived, diagnostic CT datasets which were additional to a low dose CT scan or where combined low dose and diagnostic CT data were submitted and could not be separated, and cardiac PET/CT datasets which were not for assessment of myocardial perfusion, such as multiple PET FOV localisation scans for sarcoidosis.

Studies proposing NDRLs for MI examinations have either analysed data according to dose information gathered from a population of systems (scanner types per facility) [2, 11, 15, 17], or per facility (using a dose average across all scanners at a given facility) [16]. This study analysed data according to systems as opposed to facilities, in keeping with the methodology of the other studies covering a broad range of PET/CT and SPECT/CT examinations [2, 11, 15, 17]. Lima et al. identified a possible limitation to this approach, whereby there could be a bias towards facilities with a large number of scanners, but on investigation, they found no significant influence on the distribution of doses [17].

This study has some recognised limitations. Despite this study gathering a large amount of data from 83 systems across 43 facilities, collected data were not sufficient to suggest NDRL and achievable doses for PET/CT bone and SPECT/CT sentinel node, thyroid post ablation, and octreotide/mIBG examinations. Furthermore, data were insufficient to suggest NDRLs for all clinical purposes for all examinations. Details of all CT acquisition parameters for PET/CT and SPECT/CT were collected with the intention of exploring the protocol settings contributing to the greatest dose variations, which in turn could inform dose optimisation strategies. A basic evaluation was undertaken where possible, as shown in Table 3, but a full evaluation was not feasible, due in part to differences in how scanner vendors define the reference image quality where tube current modulation is applied. This study provides suggested Nordic NDRL data for PET/CT and SPECT/CT scans. However, it is important to note that the data presented in this study are not official NDRLs, as they must first be ratified by the relevant local radiation protection authorities.

## Conclusions

This study suggests Nordic NDRL (75th percentile) and achievable dose (50th percentile) values for the CT aspect of PET/CT and SPECT/CT examinations, which are specific to the clinical purpose of CT. NDRLs are suggested for PET/CT oncology, infection/inflammation, brain and cardiac (myocardial perfusion), and SPECT/CT cardiac (myocardial perfusion), lung, bone, and parathyroid examinations. Great variations in CT doses have been identified for the same examination and clinical purpose of CT for all examinations, demonstrating great scope for optimisation. Variation in scan length has been identified as one key contributor to variation in dose for SPECT/CT examinations, and future dose optimisation efforts could focus in part, on establishing optimal scan lengths. Future publications should further communicate sources of dose variations seen in clinical practice, and how CT protocols can be optimised for PET/CT and SPECT/CT examinations. CT radiation doses delivered from PET/CT and SPECT/CT scans should change over time, for example with an increased awareness of the need for CT optimisation in MI and with greater availability of advanced scanner technologies and dose saving features. The survey should therefore be repeated in 3–5 years [18].

## Supplementary information

**Additional file 1:** Data capture booklet requesting information for each examination on scanner type, clinical purpose of CT, protocol settings and patient doses.

## Data Availability

The datasets supporting the conclusions of this article are included within the article.

## Abbreviations

AC: Attenuation correction
CT: Computed tomography
CTDI_vol_: Computed tomography dose index by volume
CTDI_vol, 75kg_: Computed tomography dose index by volume derived for a 75-kg person
DLP: Dose length product
DLP_75kg_: Dose length product derived for a 75-kg person
DRL: Diagnostic reference level
EU: European Union
FOV: Field-of-view
ICRP: International Commission on Radiological Protection
IR: Iterative reconstruction
kV: Kilovolt
mIBG: Metaiodobenzylguanidine
mA: Milliampere
mAs: Milliampere-seconds
mGy: Milligray
mGy.cm: Milligray-centimetres
MI: Molecular imaging
NDRL: National diagnostic reference level
PET: Positron emission tomography
PET/CT: Positron emission tomography/computed tomography
SIS: Sundhedsstyrelsen, Statens Institute for Strålebeskyttelse (Danish radiation protection authority)
SPECT: Single photon emission computed tomography
SPECT/CT: Single photon emission computed tomography/computed tomography
UK: United Kingdom
USA: United States of America
